# Dihydrotestosterone and 17-Estradiol Enhancement of in vitro Osteogenic Differentiation of Castrated Male Rat Bone Marrow Mesenchymal Stem Cells (rBMMSCs)

**Published:** 2019-10-01

**Authors:** FAM Abo-Aziza, AA Zaki, AS Amer, RA Lotfy

**Affiliations:** 1 Department of Parasitology and Animal Diseases, Veterinary Research Division, National Research Center, Giza, Egypt; 2 Department of Physiology, Faculty of Veterinary Medicine, Cairo University, Giza, Egypt; 3 Department of Zoology, Faculty of Women for Arts, Science and Education, Ain Shams University, Cairo, Egypt

**Keywords:** rBMMSC, Dihydrotestosterone, 17-estradiol, Osteogenesis, Castration

## Abstract

**Background:** *In vitro* impact of dihydrotestosterone (DHT) and 17-estradiol (E2) in osteogenic differentiation of castrated rat bone marrow mesenchymal stem cells (rBMMSC) still need to be clarified.

**Materials and Methods:** The viability, proliferation and density of cultured rBMMSC isolated from sham operated (Sham) and castrated (Cast) male rats were evaluated. rBMMSC were cultured with osteogenic differentiating medium (ODM) in the presence of DHT (5,10 nM) and E2 (10,100 nM). Osteogenesis was evaluated by alizarin red staining and measurement of calcium deposition and bone alkaline phosphatase (B-ALP) activity.

**Results:** Population doubling (PD) of rBMMSC isolated from Cast rats was significantly lower (P<0.05) compared to that isolated from Sham rats. rBMMSC from Cast rats showed low scattered calcified nodule after culturing in ODM and did not cause a significant increase in calcium deposition and B-ALP activity compared to rBMMSCs from Sham rats. Exposure of rBMMSC isolated from Cast rats to DHT (5 nM) or E2 (10 nM) in ODM showed medium scattered calcified nodules with significantly higher (P<0.05) calcium deposition and B-ALP activity. Moreover, exposure of rBMMSC to DHT (10 nM) or E2 (100 nM) showed high scattered calcified nodules with higher (P<0.01) calcium deposition and B-ALP activity

**Conclusion:** These results indicated that the presence of testes might participate in controlling the *in vitro *proliferation and osteogenic differentiation capacity of rBMMSCs. DHT and E2 can enhance the osteogenic capacity of rBMMSCs in a dose-dependent manner. Based on these observations, optimum usage of DHT and E2 can overcome the limitations of MSCs and advance the therapeutic bone regeneration potential in the future.

## Introduction

 Models of castration to test the function of mesenchymal stem cells (MSCs) were used mostly in preclinical prostate cancer study^1^. Although 10–15% of vertebral fractures and almost 30% of hip fractures have been recorded in men, the metabolic bone disease has been studied more intensively in women than in men^2^. Many investigations are focused on ovariectomized rats as a model of menopause to evaluate the risk of osteopenia-induced fractures^3^, while there has been a deficiency in published studies on the effect of castration^4^**.**

Rat bone marrow derived mesenchymal stem cells (rBMMSCs) are heterogeneous, pluripotent, and have multi-lineage differentiation capacity into many cell types such as osteocytes, adipocytes, chondrocytes, and muscular cells that have a major role in tissue regeneration^5^. Therefore, rBMMSCs applications have been broadly used in numerous clinical trials for the treatment of various disorders, including osteogenicdefects^6^. One of the most bone defects is osteoporosis that most frequently increased the fracture risk in the elderly^7^.

Recently,* in vivo *and *in vitro *studies on sex hormones, including testosterone and 17*β*-estradiol have a raised attention because of their effects on stem cells^8^.Testosterone and estrogen play a vital role in bone metabolism^9^. Sex differences represented by the effects of these sex steroids are usually found with bone defects^10^. Testosterone can be converted to the more potent 5-dihydrotestosterone (DHT) as it can be converted also to estrogen that has beneficial effects on bone metabolism in males through the impact on bone remodeling, including accumulation and maintenance of bone mass^11^. Therefore, the attention was lately attracted to the relation of sex hormones with the changes in bone mass and biochemical competence^12^. *In vitro *osteogenic differentiation of MSCs can be affected by several factors, including culture media, type of osteogenic media, doses and types of growth factors^13^ and the confluence at which to passage or harvest the cells^14^. Among these, it was reported that osteogenic differentiation of MSCs was achieved under the effect of several endocrine and autocrine/paracrine factors including steroids^15^. Furthermore, several studies recorded that stem cells carry estrogen and androgen receptors^16^ suggesting that these sex hormones may have an effective role in the modification of stem cells differentiation^17^. Subsequently**, **the hypogonadism-associated bone loss is firmly correlated to sex steroids reduction^11^^,^^18^. Although some *in vitro* studies reported that testosterone increases the proliferation of osteoblast progenitors, but these effects are adequately controversial^19^. It was also reported that E2 has a regulatory role of rBMMSCs expansion^20^. Interestingly, a wider range of E2 concentrations was seen to fundamentally enhance BMMSCs expansion in male rather than in female. Nonetheless, the regulatory effect of E2 on osteoblastic differentiation is still contradictory^21^.

Previous studies found that menopause women were protected by 17*β*-estradiol, and men were protected by androgensal most until 60 years^22^. However, the functional interaction of sex steroids in osteoblast differentiation and bone formation still need to be clarified. Therefore, in the present study, to test the ideality of isolation method of rBMMSCs cell counting and viability assay were done. The proliferation of isolated rBMMSCs from sham alizarin red and castrated male rats was investigated using colony forming unit-fibroblastic number (CFU-F) and population doubling (PD) assays. The effect of castration on osteogenic differentiation was studied. Finally, the effect of two doses of DHT and E2 on *in vitro *osteogenic differentiation of rBMMSCs derived from castrated rats was investigated.

## MATERIALS AND METHODS

**Ethical approval**

All procedures were performed as per to the guidelines laid down by the International Animal Ethics Committee and in accordance with the regulations stated by National Research Centre, Egypt for laboratory animals caring and use.

**Animals and surgical operation**

Twenty adult male Wistar albino rats of 70-80 days of an average weighted 150-200 gm were used throughout the study. Rats were obtained from the Laboratory Animal House, National Research Centre, Egypt. The rats were kept at 22–24°C and 50–60 % humidity. Rats were housed in two groups in the animal room and were fed standard rodent ration and water *ad libitum*. Ten healthy rats were Castrated (Cast) under diethyl ether (Sigma-Aldrich, USA) anesthesia, and all steps were made to reduce the suffering of animals. Briefly, about 3 - 4 cm straight incision was made from caudal to cranial along the scrotum under sterile conditions after pushing the testes, then spermatic cord was firmly tied and then spermatic cord was cut with scissors. The incision was closed with interrupted sutures^23^. Ten healthy rats were incised in the scrotum and the testes were pulled without removal and considered as sham operated (Sham). After surgery, all rats were housed separately and supplied with sufficient food and water available *ad libitum*. Sham and Cast rats were killed with an anesthetic overdose of diethyl ether at 21 days’ post operation. Isolated and standardized osteotomy of femur head from Sham and Cast rats were done as described previously^24^. Left femurs were used for histological examination while the right femurs were used for rBMMSCs isolation.

**Histological examination**

Microscopical features of bone trabeculae were visualized with hematoxylin-eosin stain for evaluating changes in femurs after castration. The left side of the femur of each rat was separated and fixed in 10% neutral buffered formalin (Sigma-Aldrich, USA), and then decalcified in decalcifying solution (24.4% formic acid and 0.5 N sodium hydroxide, Sigma-Aldrich, USA) for 5 days. Mixed decalcifying solution was replaced once a day for 3 days. The decalcified bones were embedded in paraffin (Sigma-Aldrich, USA), sectioned with a microtome, stained and examined as previously described^24^.

**Bone marrow collection**

Bone marrow (BM) was harvested by introducing alpha minimum essential medium (-MEM, Invitrogen, USA) in the collected right femur and flushing the contents. BM was diluted with -MEM for rBMMSCsisolation^24^.

**Isolation and cultivation of rBMMSCs**

Mononuclear cells (MNCs) isolation was performed by gradient centrifugation of 2 ml BM blood on the same volume of Ficoll- Histopaque®-1077 (Sigma-Aldrich, USA) for 20 minutes at 2000 rpm and room temperature^25^. MNCs were then collected, washed twice and resuspended in -MEM. The cells were counted using hemocytometer and 1 × 10^6 ^BM derived all nucleated cells were seeded into 100 mm culture plates (Corning, USA) and incubated to adhere at 37°C in humidified atmosphere with 5% CO2. Two days later, the media was changed to discard the non-adherent cells, while the adhered cells were kept in -MEM (Invitrogen, USA) supplemented with 20% fetal bovine serum (FBS, Lonza, Switzerland), 55 μM 2-mercaptoethanol (Lonza, Switzerland), 2 mM L-glutamine (Lonza, Switzerland) and penicillin/streptomycin (Lonza, Switzerland)^24^. Changing of the media was done twice a week and the plates were microscopically observed. After the colonies reached 80% confluence, the cells were harvested after detachment using1 ml trypsin-EDTA 0.25%,(Lonza, Switzerland) for each plate and incubated for 5 min at 37°C in humidified atmosphere with 5% CO2. Then, -MEM supplemented with 20% FBS was added for trypsin deactivation. The detached cells were collected in a sterile tube and centrifuged at 2000 rpm for 5 min. The pelleted cells were resuspended with 1ml -MEM and 0.25 x 10^6 ^cells were cultured in T-25 flask (Nunc, USA) and designated as passage 1 to be exposed for cell proliferation and differentiation assays.

**Cell counting**

After two weeks, the third passage of rBMMSCs derived from Sham and Cast rats were washed twice with phosphate buffer saline (PBS, PH 7.4). The cells were detached using trypsin-EDTA 0.25%, then collected and centrifuged at 2000 rpm for 5 min. The cells were suspended in 2ml -MEM, and their number was counted in an aliquot using hemocytometer.

**Cell viability assay**

Upon reaching 80% confluence, the viability of rBMMSCs derived from Sham and Cast rats were evaluated and the dead cells were excluded using trypan blue stain (Sigma-Aldrich, USA) ^24^^. ^The dead cells were stained blue, while the live cells remained unstained.

**Cell proliferation assay**

To determine the proliferation capacity of rBMMSCs derived from Sham and Cast rats, CFU-F assay and PD were performed.

**CFU-F assay**

To determine CFU-F, 1 x 10^6^ cells from the third passage of rBMMSCs derived from Sham and Cast rats were seeded on 100 mm culture dish. Upon reaching 80% confluence, approximately after 14 days, the cultured cells were washed with PBS, PH 7.4 and stained with 1% toluidine blue solution (Sigma-Aldrich, USA) in 2% paraformaldehyde (Sigma-Aldrich, USA). The cells were then examined under inverted microscope, and each cell group containing in excess of 50 cells was counted as colony^26^.

**PD**

To determine the proliferation capacity of cultured rBMMSCs derived from Sham and Cast rats, PD assay was calculated^27^. Briefly, the cells were detached using trypsin-EDTA 0.25%, and a total of 0.25 × 10^6^ cells from each group were seeded on culture flasks. Upon reaching 80% confluence, the cells were detached using trypsin-EDTA, and then passaged again at the same cell density. The cells were monitored, and the number was counted at every passage. PD in each passage was calculated using the following equation: log_2_ (number of harvested cells / number of plated cells). Then, the final PD was calculated by accumulative addition of total numbers of each passage until the stopping of cell division. 

**Effect of castration on** ***in vitro*** **osteogenic differentiation**

 The confluent rBMMSCs of Sham and Cast rats of passage 3 were cultured under osteogenic culture conditions for 3-4 weeks at 37°C in humidified atmosphere with 5% CO2 with changing the medium twice a week^28^. The osteogenic differentiation medium (ODM) was -MEM supplemented with 20% FBS, 2 mML-glutamine, 55 M 2-mercaptoethanol, 100 M L-ascorbic acid 2-phosphate (Sigma-Aldrich, USA), 2 mM-glycerophosphate (Sigma-Aldrich, USA), dexamethasone(Sigma-Aldrich, USA) and 10 nMpenicillin/ streptomycin.

**Sex steroids supplementation**

 rBMMSCs derived from Sham rats were cultured with ODM (Sham +ODM). rBMMSCs derived from Cast rats were exposed to OMD supplemented with different concentrations of DHT (Sigma-Aldrich, USA) and E2 (Sigma-Aldrich, USA) as follow: (1) rBMMSCs derived from Cast were cultured with ODM(Cast +ODM), (2) rBMMSCs derived from Cast were cultured with ODM supplemented with 5 nM of DHT (Cast +ODM+5nMDHT), (3) rBMMSCs derived from Cast were cultured with ODMs supplemented with 10 nMDHT (Cast +ODM10nMDHT), (4) rBMMSCs derived from Cast were cultured with ODM supplemented with 10 nM E2(Cast +ODM+10nM E2), (5) rBMMSCs derived from Cast were cultured with ODM supplemented with 100 nME2 (Cast +ODM+100nM E2). These concentrations were chosen to evaluate the effects of DHT and E2 at low and high concentrations based upon previous dose-response studies^21^.The medium was changed twice a week and osteogenesis confirmation was done using alizarin red stain and measurement of calcium deposition and bone ALP (B-ALP) activity.

**Evaluation of osteogenic differentiation**

**Alizarin red staining**

To highlight extracellular mineralization, the osteogenic- induced cells were stained as described previously^28^. Briefly, washing of osteogenic-induced cells with PBS was done followed by fixation in 60% (v/v) isopropanol (Sigma-Aldrich, USA) for one min in 60mm dishes. The cells were rehydrated using distilled water and stained with 1% alizarin red stain, PH 4.1 (Sigma-Aldrich, USA). The cells were washed four times with distilled water after 3 min incubation at room temperature, and then they were left in the air to dry. Finally, the mineralized nodules were imaged using inverted microscope.

**Calcium deposition assay**

To determine osteoblastic differentiation, calcium assay was performed^29^. Seeding a fixed number of all differentiated cells into the wells was done. The cells were washed twice with PBS and extracted off the wells in 0.5 N HCl (Sigma-Aldrich, USA). The accumulated calcium was removed from the cellular component by 5 h shaking at 4^◦^C, then centrifuged at 2,000 rpm for 10 min. Calcium was determined in the supernatant using calcium colorimetric assay kit (Abcam, USA). Total calcium was determined by the measurement of absorbance at 575 nm and calculated from parallel prepared standard solutions and expressed as mg/well.

**B-ALP activity measurement**

Osteogenic differentiation in each group was evaluated by B-ALP activity assay as described previously^30^. Osteogenic-induced cells were incubated for 5 min at room temperature with 20 𝜇L/well of 0.1% Triton X-100 (Sigma-Aldrich, USA) for cell lysis. B-ALP assay kit (Abcam, USA) was used to measure B-ALP activity of the cell lysates at absorbance 405 nm using a Microplate Reader.

**Statistical analysis**

Data analysis was performed using the statistical software of Statistical Package for the Social Sciences version 19 (SPSS-19). Correlation analysis was used to obtain P values and Pearson coefficient.

## Results

**Figure 1:**
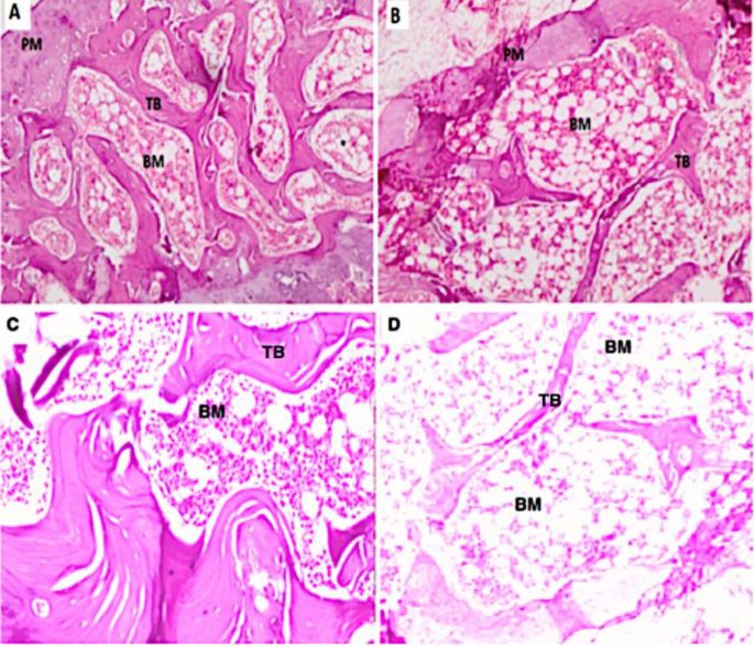
Histological profiles of cortical regions of the left femur. A, C for Sham group; B, D. for castrated group. All micrographs were visualized by hematoxylin-eosin staining (A, B: ×40, C,D: x100). Trabecular bone (TB) and bone marrow (BM) are differentially stained and periosteum (PM) is noticed as the outmost layer of bone (A). Sponge bone showed uniform thinning of the trabeculae in the castrated rats with wide intertrabecular spaces (B). Sham rats showed normal cancellous bone trabeculae with red bone marrow (C). After castration, cancellous bone trabeculae appeared as discontinuous bony ossicles separated by widened fatty bone marrow spaces (D).The bar indicates 100 mm.

 The architecture of selected sections of femoral diaphysis for sham and castrated rats can be characterized by the number and thickness of trabeculae (TB) and intertrabecular spaces (Figure 1). Histological image of cancellous bone showed uniform thinning of the trabeculaein the castrated rats resulting in widening of intertrabecular spaces in the femur head (Figure 1B). After castration, thin cancellous bone trabeculae appeared as discontinuous bony ossicles separated by wide fatty bone marrow spaces (Figure 1D).

**Figure 2:**
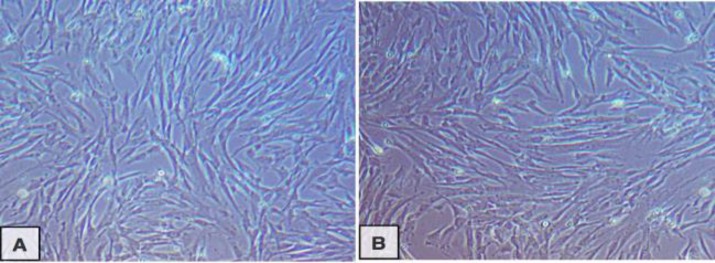
Photomicrograph image of cultured BMMSCs of Sham (A) and Cast rats (B) reached 80% confluence after 15 and 17days respectively(X20).

Inverted microscopical examination showed that rBMMSCs of Sham and Cast rats were isolated from BM and adhered on the culture dishes bottom. After approximately one to two weeks, rBMMSCs from Sham and Castrates continued to proliferate and propagate until reached 80% confluence. The cells in the two groups became spindle in shape with process and appeared as fibroblast, but rBMMSCs derived from Cast rats took more long time to reach 80% confluence than that derived from Sham rats as observed by inverted microscope (Figure 2). 

**Cell viability**

**Figure 3:**
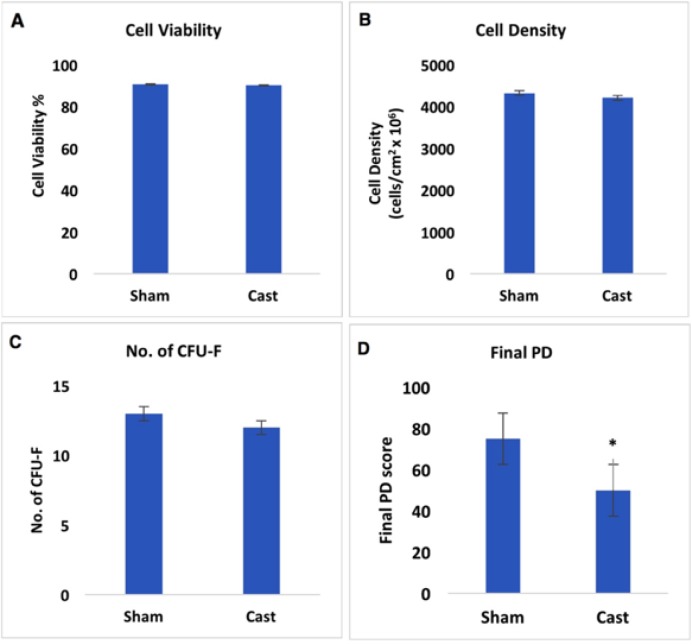
Effect of castration on viability, density, no. of CFU-F and PD of rBMMSCS derived from Sham and Cast rats**(A) **The viability of rBMMSCs was more than 90% in both Sham (90.74%) and Cast (90.23%).**(B)**Cell counting showed the cell density of rBMMSCs isolated fromSham (4.325 x10^6^ cells/cm^2^) and Cast (4.21210^6^ cells/cm^2^). **(C) **The number of CFU-F from rBMMSCsof Sham and Cast rats upon reaching 80% confluence. **(D)**rBMMSCs derived from Cast rats exhibit a significant lower PD final score when compared to that from Sham rats.Error bars refer to Mean ± SE. * indicate significant at P<0.05 comparing to non-marked groups.

The viability of rBMMSCS was more than 90% at 80% confluence. There was no significant difference in the viability of cells from Sham (90.74%) and Cast (90.23%) rats (Figure 3-A).

**Cell density**

 rBMMSCs were isolated from Sham and Cast rats, then cultured and the cell densities per cm^2^ culture area were calculated after 2 weeks of incubation. It was found that the cell density of rBMMSCs from Cast rats (4.212x10^4^ cells/cm^2^) was not significantly different than that of Sham rats (4.325x10^4^ cells/cm^2^) (Figure 3-B).

**CFU-F**

When rBMMSCs reached 80% confluence, they became strongly attached to the extracellular matrix and highly tended to form CFU-Fit. No significant difference was recorded between the CFU-F number of rBMMSCs from Sham and Cast rats (Figure 3-C).

 ‬‬‬‬‬‬‬‬‬‬‬‬‬‬‬‬‬‬‬‬‬‬‬‬‬‬‬‬‬‬‬‬‬

**PD**

Final PD score determined the maximal potential of proliferation of cultured cells. The results showed that rBMMSCs derived from Cast rats exhibited a significantly lower final PD when compared to that from Sham rats (Figure 3-D). 

**Osteogenic differentiation**

**Figure 4:**
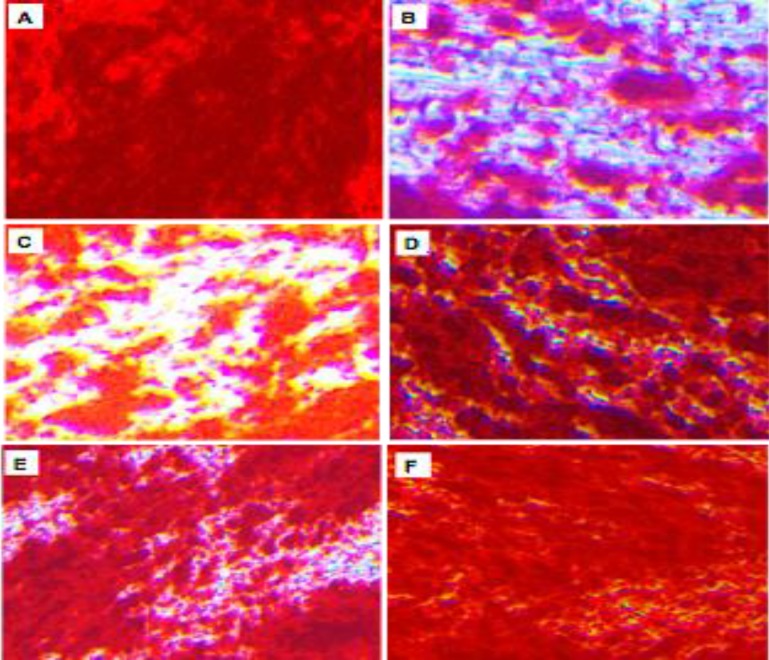
Photomicrograph of alizarin red staining of* in vitro *osteogenic differentiation of rBMMSCs**. **Sham +ODM showed highly scattered red calcified nodules** (A).**Cast +OMD showed low scattered red calcified nodules** (B)**. Cast +OMD+5nMDHT showed medium scattered red calcified nodules**(C)**. Cast +OMD+10nMDHT showed high scattered red calcified nodules **(D).**Cast +OMD+10nM E2showing medium scattered red calcified nodules **(E).**Cast + OMD+100 nME2showing high scattered red calcified nodules **(F).**

**Figure 5:**
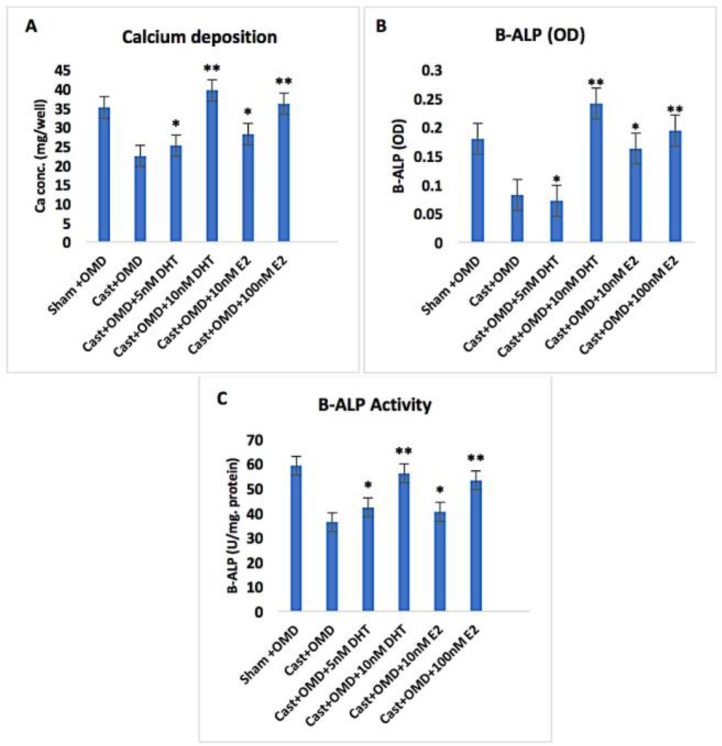
*In vitro*osteogenic differentiated rBMMSCs derived fromCast+OMD+5nM DHT, Cast+OMD+10nMDHT, Cast +OMD+10nM E2, Cast +OMD+100nM E2 groups showed a significant elevationin calcium deposition (mg/well) andB-ALP(U/mg protein)activity. Error bars refer to Mean ± SE. * and ** indicate significant at P<0.05 and P<0.01 respectively comparing to non-marked groups.

To assess the effect of castration on osteogenic differentiation of rBMMSCs, induction of *in vitro *osteogenic differentiation of rBMMSCs derived from Sham and Castrates was done and stained using the alizarin red method, as well as calcium deposition and B-ALP activity were determined. Sham +ODM showed highly scattered red calcified nodules (Figure 4-A). However, Cast +ODM showed low scattered red calcified nodules (Figure 4-B) and did not cause a significant increase in calcium deposition and B-ALP activity compared to Sham + ODM(Figure 5). 

Culturing of Cast +ODM +5nM DHT showed medium scattered red calcified nodules (Figure 4-C). However, these cells showed significantly higher calcium deposition (25.17 mg/well) and B-ALP activity (42.27 U/mg protein) at P<0.05 compared to that of Cast +ODM (Figure 5). Moreover, high dose of DHT (10nM) supplementation in ODM in Cast +ODM+10nM DHT showed high scattered red calcified nodules (Figure 4-D). Likewise, Cast +ODM +10nM DHT showed significantly higher calcium deposition (39.62 mg/well) and B-ALP activity (56.12 U/mg protein) at P<0.01 compared to that of Cast +ODM (Figure 5). 

**Figure 6:**
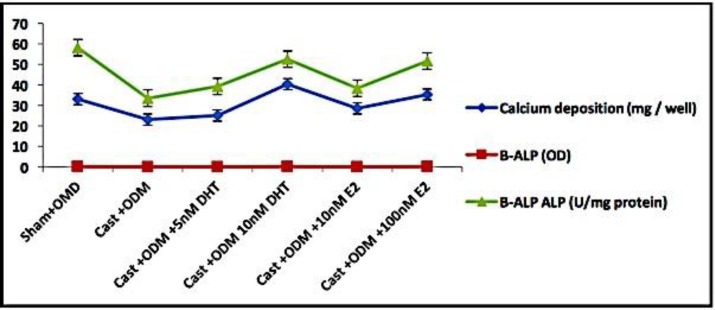
Correlation of calcium deposition (mg / well), B-ALP (OD) and B-ALP ALP (U/mg protein) of rBMMSCs isolated from different groups studied.

Furthermore, Cast +ODM +10nME2 showed medium scattered red calcified nodules by alizarin red staining (Figure 4-E). Moreover, osteogenic differentiation of Cast +ODM +100nM E2 showed high scattered red calcified nodules by alizarin red staining (Figure 4-F). In addition, ODM supplemented with 10 nM of E2 significantly increases calcium deposition (28.18 mg/well) and B-ALP (40.41 U/mg protein) at P<0.05 as well as 100 nM supplementation of E2 significantly increase calcium deposition (36.12 mg/well) and B-ALP (35.28 U/mg protein) at P<0.01 (Figure 5). The correlation of calcium deposition (mg / well), B-ALP (OD) and B-ALP ALP (U/mg protein) of rBMMSCs isolated from different groups was also done (Figure 6).

Calcium and B-ALP concentrations were showed significant Pearson correlations. It was recorded that Pearson correlation of calcium and B-ALP (U/mg protein) was 0.816 at P= 0.092, while the correlation of calcium and B-ALP (OD) was 0.956 at P= 0.011. However, Pearson correlation of B-ALP (OD) and B-ALP ALP (U/mg protein) was 0.746 at P= 0.147.

## Discussion

 Recent research is moving away from embryonic stem cells (ESCs) due to ethics surrounding them. Laboratory attention was concentrated on stem cells derived from adult tissues. However, rBMMSCs collect the basic characteristics of stem cells, including the ability to self-renew and differentiate into multiple cell types. Commonly, the three important features allowing rBMMSCs to be isolated from various species are the plastic surfaces adherent ability, the high proliferation capacity, and the greater vulnerability to trypsin digestion compared to the other BM cells^31^. Therefore, BMMSC was used in the present study. 

rBMMSCs undergo proliferation in response to various factors. These factors may be related to the culturing process such as the optimum confluence at which to passage or harvest the cells^14^ or may be due to the donor-related effect such as sex steroids^32^. Although rBMMSCs are promising cell types that widely investigated for their clinical uses, their reduced number in the body are usually coupled with various dysfunctions^22^. Since sex steroids have a thoughtful impact on different cell types, the present study investigated the proliferation of rBMMSCs of castrated male rats. 

The viability of rBMMSCS was more than 90% at 80% confluence. There was no significant difference in the viability of cells from Sham (90.74%) and Cast (90.23%) rats. It was found that the cell density of rBMMSCs from Cast rats (4.212x10^4^ cells/cm^2^) was not significantly different from that of Sham rats (4.325x10^4^ cells/cm^2^). These results are consistent with the results obtained by Al-Mutairi et al^33^**.** This data collectively indicated that castration did not affect the viability and the cell density of rBMMSCs, and the variation of the effect of castration may involve the advanced process. In the present study, it was found that rBMMSCs from Sham and Cast rats continued to proliferate and propagate until they reached 80% confluence approximately after 15 and 17 days, respectively.This result was parallel to the previous study where BMMSC isolated from sham and ovariectomized rats reached 80% confluences nearly at the same time^33^. In this investigation, the cells in the two groups became spindle in shape with process and appeared as fibroblast as observed by inverted microscope. *In vitro* preparations of rBMMSCs are defined as a mixture of heterogenic adhered growing cells having similar spindle-shaped morphology^34^. Several studies have reported that *in vitro* cultured rBMMSCs are mostly distinct subpopulations, and the minor of expanded MSCs colonies have a full proliferation potentiality^35^, referring to functional variances of BMMSC subpopulations. It was indicated in previous studies that female sex hormone, estrogen, was shared in the control of MSCs proliferation^20^. This effect was previously characterized in stem cells derived from both male and female BM^36^. 

For osteogenesis, the media used in the present study contained substances considered as stimuli such as ascorbic acid, β-glycerphosphate (β-GP) and dexamethasone. Ascorbic acid actively induced mineralization and promoted collagen synthesis. β-glycerphosphate involved in the construction of mineral hydroxyapatite within the collagenous matrix^37^. In addition, ascorbic acid prompted ALP activity, where it split phosphate from the added β-GP, phosphate move in the cell via Na^2+^ transporters^38^. In this study, to assess whether castration affected osteogenic differentiation of rBMMSCs,* in vitro* osteogenic differentiation of Sham and Cast rats derived rBMMSCs was induced and stained with alizarin red staining. Moreover, calcium deposition and B-ALP activity were measured. Sham +ODM showed highly scattered red calcified nodules. However, Cast +ODM showed low scattered red calcified nodules and did not cause a significant increase in calcium deposition and B-ALP activity compared to Sham + ODM. These results are in agreement with the findings of Al-Mutairi et al.^33^ in ovariectomized rats. In males, the skeletal integrity maintenance is androgen dependent and inherited or acquired testicular function disorders are usually coupled with bone loss^39^. Definitely, congenital hypogonadism in male prompts osteoporosis that can be avoided by administering testosterone^40^. Likewise, castration could lead to osteopenia as a result of elevated bone resorption^41^. In rats, orchidectomy-induced bone loss has resulted from elevated endosteal bone resorption, trabecular bone loss, and increased number of osteoclasts^42^.These alterations can be reversed by the administration of testosterone or dihydrotestosterone (DHT)^43^. In addition, the detection of the receptors of both androgen and estrogen in bone^44^, and the *in vitro *conversion of testosterone into both dihydrotestosterone and estrogen in the bone of rat^45^and human^46^ suggested that these steroids have direct potential effects on bone.

Culturing of Cast +ODM +5nM DHT showed medium scattered red calcified nodules. However, these cells showed significantly higher calcium deposition and B-ALP activity compared to that of Cast +ODM. Moreover, high dose of DHT (10nM) supplementation in ODM showed high scattered red calcified nodules. Likewise, it showed significantly higher calcium deposition and B-ALP activity as shown in Cast +ODM +10nM DHT compared to that of Cast +ODM. Furthermore, Cast +ODM +10nM E2 showed medium scattered red calcified nodules by alizarin red staining. Moreover, osteogenic differentiation of Cast +ODM +100nM E2 showed highly scattered red calcified nodules by alizarin red staining. In addition, ODM supplemented with 10 nM of E2 significantly increased calcium deposition and B-ALP and 100 nM supplementation of E2 significantly increased calcium deposition and B-ALP. Concerning the role of estrogens, there is an indication that 17β-estradiol supports osteogenic differentiation of MSCs mainly via ERα receptor^47^^,^^48^. This preference of the receptor can be referred to sex differences and individual variation of osteoblast responses of MSCs to estrogen demonstrated by ERα polymorphism^49^. Supplementation of 17β-estradiol to osteogenic differentiation medium of rBMMSCs can increase bone morphogenetic protein (BMP) and osteocalcin expression and significantly increase calcium deposition^50^. Estrogen also maintains bone density by suppressing bone resorption and enhancing bone formation as reported recently^51^. This effect might be through their nuclear receptors^52^. Martin et al.^53^ indicated that estrogen has an important effect in regulating differentiation of osteoblasts. Estrogen administration to ovariectomized rodent was accompanied by decrease in apoptosis of MSCs in vitro^54^. In the *in vitro* studies**, **the osteogenic differentiation of rBMMSCs was increased when estrogen was added to the media as a result of the elevation of bone morphogenetic protein expression and significant increase of calcium and ALP deposition^50^. Previous studies discussed how estrogen and testosterone direct the differentiation, suggesting that the effect of estrogen on their receptors might affect the function of those cells^17^. On the other hand, the function of testosterone on stem cells remains debatable. Some studies suggested that testosterone might inhibit the functions of stem cell, and anti-testosterone could reflex these effects^16^. Thus, modulation of stem cells functions via sex hormones may be the goal of recent therapeutic efficacy^55^. The molecular mechanism by which steroids enhance proliferation was previously mentioned by activating Notch and JNK signaling pathway and upregulation of ERs transcripts activation^56^^,^^57^. However, estrogens are critical for the maintenance of bone mass in women, androgen level decrease can also lead to bone loss in men^11^. Therefore, testosterone replacement is useful for bone mineral density (BMD) maintenance^18^, in which both the effects of the androgen and estrogen converted from the androgen are involved^11^. 

Testosterone to some extent differed from estrogen in previous literature. Osteogenesis was enhanced by androgen through activation of estrogen receptors via Akt activation as reported^58^or through decrease insulin like growth factor-I^59^ that are a key regulator of androgen-mediated osteoblast differentiation. Different genomic and non-genomic pathways may be also involved in mediating these effects. The non-genomic pathways are enhanced through stimulation of src kinase by Akt activation^60^^,^^61^. Testosterone administration can also activate MAP kinase signaling cascade leading to increase the expression of Raf-1 and ERK-2^60^.

This study emphasizes the importance to understand the distinctive effects of sex hormones and the absence of testes on bone metabolism. The availability of MSCs from multiple body sources including bone marrow and the properly simple *in vitro* propagation of the cells to the desired cell type in addition to the lack of ethical problems have made these cells a favorite source for stem cell therapy and effectively overcome the limitations of MSCs. The obtained data indicated that the presence of testes might participate in controlling the *in vitro *BMMSCs proliferation and osteogenic differentiation. Treatment of MSCs with DHT and E2 can enhance osteogenic capacity in a dose-dependent manner. The optimal application of sex hormones can develop the therapeutic potential of MSCs in applied bone regeneration medical practice in the future.

## COMPETING INTERESTS

 The authors declare that they have no competing interests. 
